# Feasibility and cost description of highly intensive rehabilitation involving new technologies in patients with post-acute stroke—a trial of the Swiss RehabTech Initiative

**DOI:** 10.1186/s40814-022-01086-0

**Published:** 2022-07-05

**Authors:** Corina Schuster-Amft, Jan Kool, J. Carsten Möller, Raoul Schweinfurther, Markus J. Ernst, Leah Reicherzer, Carina Ziller, Martin E. Schwab, Simon Wieser, Markus Wirz, for the SRTI study group, Alexandra Menig, Liliana P. Paredes, Heike Rosemeier

**Affiliations:** 1 Research Department, Reha Rheinfelden, Rheinfelden, Switzerland; 2 School of Engineering and Computer Science, Bern University of Applied Sciences, Biel, Switzerland; 3 Department of Sports, Exercise and Health, University of Basel, Basel, Switzerland; 4 Rehabilitation Centre Valens, Valens, Switzerland; 5 Center for Neurological Rehabilitation, Zihlschlacht, Switzerland; 6 Faculty of Medicine, Philipps University, Marburg, Germany; 7 ZHAW Zurich University of Applied Sciences, Institute of Physiotherapy, Katharina-Sulzer-Platz 9, Postfach, CH-8401 Winterthur, Switzerland; 8 Institute for Regenerative Medicine, University of Zurich, Zurich, Switzerland; 9 ZHAW Zurich University of Applied Sciences, Winterthur Institute of Health Economics, Winterthur, Switzerland

**Keywords:** Stroke rehabilitation (MeSH), Technology-assisted training, High intensity, Physical exertion (MeSH)

## Abstract

**Background:**

There is a need to provide highly repetitive and intensive therapy programs for patients after stroke to improve sensorimotor impairment. The employment of technology-assisted training may facilitate access to individualized rehabilitation of high intensity. The purpose of this study was to evaluate the safety and acceptance of a high-intensity technology-assisted training for patients after stroke in the subacute or chronic phase and to establish its feasibility for a subsequent randomized controlled trial.

**Methods:**

A longitudinal, multi-center, single-group study was conducted in four rehabilitation clinics. Patients participated in a high-intensity 4-week technology-assisted trainings consisting of 3 to 5 training days per week and at least 5 training sessions per day with a duration of 45 min each. Feasibility was evaluated by examining recruitment, intervention-related outcomes (adherence, subjectively perceived effort and effectiveness, adverse events), patient-related outcomes, and efficiency gains. Secondary outcomes focused on all three domains of the International Classification of Functioning Disability and Health. Data were analyzed and presented in a descriptive manner.

**Results:**

In total, 14 patients after stroke were included. Participants exercised between 12 and 21 days and received between 28 and 82 (mean 46 ± 15) technology-assisted trainings during the study period, which corresponded to 2 to 7 daily interventions. Treatment was safe. No serious adverse events were reported. Minor adverse events were related to tiredness and exertion. From baseline to the end of the intervention, patients improved in several functional performance assessments of the upper and lower extremities. The efficiency gains of the trainings amounted to 10% to 58%, in particular for training of the whole body and for walking training in severely impaired patients.

**Conclusions:**

Highly intensive technology-assisted training appears to be feasible for in- and outpatients in the subacute or chronic phase after stroke. Further clinical trials are warranted in order to define the most comprehensive approach to highly intensive technology-assisted training and to investigate its efficacy in patients with neurological disorders.

**Trial registration:**

ClinicalTrials.gov Identifier: NCT03641651 at August 31st 2018

## Key messages regarding feasibility

- What uncertainties existed regarding the feasibility? Do patients after stroke tolerate highly intensive trainings and how can such extraordinary long trainings be implemented?
- What are the key feasibility findings? Highly intensive technology-supported training in general is feasible. However, it seems to be feasible only for a subset of patients after stroke with residual functional limitations, only minor cognitive impairment, and a health status allowing to tolerate the training load.
- What are the implications of the feasibility findings for the design of the main study? Inclusion and exclusion criteria might be less restrictive. The logistics around the training must be taken into account, e.g., how do patients come to the training facility.

## Background

Stroke is the second leading cause of death worldwide with 5.5 million deaths in 2016. With more than 80 million stroke survivors worldwide, stroke is a major cause of disability. In spite of declining incidence, improved survival, population growth, and aging may lead to an increase of people living with a stroke [1]. Of all stroke survivors, 20% remain dependent in their mobility [2]. In Europe, the total costs resulting from strokes were 37.4 billion in 2010 [1]. In Switzerland, in 2019, 19.2% of healthcare costs were spent on acute inpatient treatment, while only 2.2% were spent on inpatient rehabilitation and 2.5% for outpatient rehabilitation including physical and occupational therapy [3].

Highly intensive exercise-based rehabilitation improves patient outcomes. There is strong evidence that more intensive training leads to better rehabilitation outcomes in individuals with stroke [4–8] and reduces hospital readmission rates [9]. However, highly intensive exercise-based rehabilitation reported in studies is not generally available. During rehabilitation, individuals post-stroke make significantly less use of their more affected (− 60%) and less affected arm (− 30%) compared with healthy persons [10]. This non-use leads to further impairment [11]. In addition, therapy time during a regular day at a rehabilitation hospital is generally limited, mostly due to management decisions, lack of structured organization, and inefficient use of resources [12]. In four European rehabilitation centers, the amount of therapy offered to individuals with stroke varied considerably from 1 to 3 h per day. Here, less therapy time per day was associated with lower functional recovery [13]. Other research showed that only 4 min of patient activity and as little as 23–32 repetitions were observed during an activity-related arm therapy session [14, 15] and 357 repetitions during a lower extremity therapy session. The awareness that rehabilitation outcome improves with increasing therapy dose has influenced health insurance reimbursement in Switzerland. From January 2022 on, inpatient rehabilitation will only be reimbursed, if patients attend a minimum of 450 min of therapy per week [16]. The increased use of rehabilitation technologies allows one therapist to supervise several patients, thereby offering higher treatment intensity without increasing staff costs.

Discharge from inpatient rehabilitation is usually planned as soon as no inpatient care is needed, on average within the first 6 weeks after stroke which is long before patients reach full recovery. Compared with inpatient rehabilitation, the intensity of outpatient rehabilitation intensity is considerably lower. Reaching the full recovery potential is further threatened by the reduction of the length of stay for inpatient rehabilitation in Switzerland, which has decreased by 12% from 27.1 days in 2002 to 23.8 days in 2019 [17]. As a consequence, many individuals are discharged from rehabilitation considerably short of attaining their optimal potential. In conclusion, more training is required during and after inpatient rehabilitation to exploit the full potential of recovery.

Technology enables robot-assisted and electromechanical gait and arm training, here referred to as technology-assisted-training (TAT). The devices reduce therapists’ physical burden thereby allowing longer training. Exhaustion of the therapist does not limit training duration. After the implementation of such devices, participants performed on average 569 functional arm movements more per session [18] and allowed walking distances of up to 2000 m or 3300 steps within a session [19].

Systematic reviews report positive effects of TAT for gait [20] and upper extremity function [21, 22]. One out of eight gait dependencies could be prevented if patients received TAT for improving walking function in addition to their regular therapy program [20]. More intensive gait TAT programs lead to better outcomes (gait: [20]; arm: [23]). Arm TAT improves activities of daily living and arm function in individuals post-stroke [24, 25].

Even though reviews report comparable effects of arm TAT if compared with conventional therapy [24, 25], TAT is more cost-efficient if one therapist supervises several patients in parallel.

Availability of TAT is still very sparse in outpatient rehabilitation settings though it would support patients exploiting their full potential of recovery. Improving the availability needs overcoming the barriers related to restrictions in healthcare insurance reimbursement.

Randomized controlled trials in outpatient settings are needed to evaluate the cost-effectiveness of highly intensive TAT to improve arm function and gait in patients after a stroke (PaS).

The Swiss RehabTech Initiative (SRTI) is an interest group with the aim to foster the meaningful implementation of technologies into the rehabilitation path, to promote the creation of appropriate reimbursement schemes and to facilitate the inclusion of rehabilitation technologies into the curricula of therapeutic professions.

Since highly intensive TAT is a complex intervention with a number of unknown elements, the present study focuses on the feasibility of a future full-scale randomized controlled trial (RCT) [26]. Such an explanatory trial would be based on multiple centers; therefore, we also planned this feasibility study in four centers. Our objectives were to evaluate (a) recruitment rate, reasons for exclusion, and possibilities to improve recruitment rate; (b) feasibility for centers to follow the standardized treatment protocol for the complex highly intensive technology-assisted rehabilitation intervention; (c) patients’ and therapists’ acceptance of the intervention; and (d) adverse effects of highly intensive TAT. In our study, we focused on highly intensive TAT. Consequently, estimating the effect of highly intensive TAT compared with usual low-dose outpatient treatment was not expected to be conclusive.

## Methods

### Study design, setting, and ethical approval

A multicenter single-arm feasibility trial was initiated by the SRTI in preparation for a RCT. For that purpose, a longitudinal single-group design was chosen. The study was implemented at four neurological rehabilitation centers across the German-speaking part of Switzerland, Reha Rheinfelden, Kliniken Valens, Klinik Lengg, and Rehaklinik Zihlschlacht, and conducted in accordance with the Declaration of Helsinki. Ethical approval was obtained from the lead ethical committee of Canton Zurich (BASEC-nr: 2018-01214) with the involvement of the ethical committees of Eastern Switzerland and Northwest and Central Switzerland. The study was registered with clinicalTrials.gov (NCT 03641651).

Our reporting follows the CONSORT 2010 extension guideline on randomized pilot and feasibility trials [27].

### Patients and recruitment

Patients were eligible when they fulfilled the following inclusion criteria:

- Patients with residual hemiparesis after cerebrovascular accident
- Up to 12 months after the event
- Primary rehabilitation terminated
- Able to cognitively comprehend the aim of the project corresponding to equal or more than 22 out of 30 points of the Montreal Cognitive Assessment (MoCA) [28]
- General health condition allows for intensive rehabilitative training with limited supervision, i.e., clearance of responsible physician
- Understand written and spoken German language

Patients presenting with any contraindication for the training with the respective devices were not considered for participation.

After study start, all sites recognized recruitment difficulties. Therefore, we amended the protocol and changed the inclusion criteria to include PaS without a restriction on the time after the stroke.

For this feasibility trial, it was planned to recruit five patients at each trial site. Recruitment strategies differed across the study sites: in-house patient database screening, flyer distribution, and direct contact by clinical staff. After a screening visit, written informed consent was obtained before data collection. After consultation of the responsible disciplines (e.g., therapists), the clearance for intensive training of the respective physician was obtained to confirm eligibility of the participant.

### Intervention

The intervention consisted of a highly intensive four-week TAT in addition to usual care. TAT took place in an outpatient or inpatient setting. Table 1 provides an overview on the interventions based on the Template for Intervention Description and Replication (TIDieR, Table 2 [29];.

**Table 1:** TIDieR checklist for intervention description

Brief name	Highly intensive technology-assisted training (TAT) involving new technologies
Why	Technological devices were chosen with the prerequisite to provide feedback and allow a targeted, intensive, and dense training.
What (materials/procedures)	A broad range of available technological systems were used in the intervention, including VR-based, electromechanically assisted and sensor-based devices for gait, upper, and lower extremity training. The selection of therapy devices depended on the impairments of the patient and resources of the clinic. The following devices were used (in alphabetical order): Allegro Medical Device (Dynamic Devices, Zürich, Switzerland), Amadeo (tyromotion, Graz, Austria), Andago (Hocoma AG, Volketswil, Switzerland), Armeo Boom, Armeo Power and Armeo Spring (Hocoma AG, Volketswil, Switzerland), Bi-Manu-Trainer (Reha-Stim Medtec AG, Schlieren, Switzerland), C-Mill (c-mill-technologie AG, Port, Switzerland), EksoGT (EkSo Bionics, Richmond, USA), Erigo (Hocoma AG, Volketswil, Switzerland), FLOAT (Reha-Stim Medtec AG, Schlieren, Switzerland), Gloreha (Idrogenet srl, Lumezzane, Italy), HAL (Cyberdyne Inc, Tsukuba, Japan), Lokomat (Hocoma AG, Volketswil, Switzerland), mindmaze (MindMaze, Lausanne, Switzerland), MOTOmed (Reck, Betzenweiler, Germany), Myro (tyromotion, Graz, Austria), NuStep (NuStep LLC, Ann Arbor, USA), and Valedo Motion (Hocoma AG, Volketswil, Switzerland)
Who provided	The training was supervised or guided by an officially recognized physiotherapist, occupational therapist, or sports scientist/therapist holding a BSc or MSc degree. All therapists were specially trained in the use of the devices and experienced in treating neurological patients.
How	The ratio of patient to therapist was between 1:1 and 3:1. Safety for patients was ensured at all time.
Where	The training took place in one of the four participating rehabilitation centers.
When and how much	A training series lasted 4 weeks and comprised 3 to 5 training days per week. One training day included at least five trainings with a duration of 45 min per training.
Tailoring	Patients’ goals and preferences were incorporated. Patients received a tailored training plan based on the patient’s needs, impairments, and preferences. Absolute and relative contraindications for the training with any of the respective devices were considered. Training sessions were intensified by increasing exercise duration, resistance or complexity of the exercise.
Modifications	A maximum training break of 7 days was tolerated. If the maximal program (5 sessions/day) could not be tolerated by patients or due to scheduling reasons, a reduction of training intensity in terms of days, sessions/day, and/or duration of a session was considered.

**Table 2:** Comparison of required staff and time between therapeutic trainings without (reference) and with devices for the calculation of efficiency gains

Training	Reference/device	Therapists required	Additional preparatory time
Walking in severely impaired patients	Reference	2	10
	Lokomat	0.5	15
	Ekso/HAL	1	15
Walking in moderately to mildly impaired patients	Reference	1	5
	C-Mill/Treadmill/Andago	1	5
	FLOAT	0.5	10
Lower extremity	Reference	1	0
	Allegro	0.5	10
	Motomed LE	0.3	10
Upper extremity	Reference	1	5
	Armeo/Pablo	0.5	10
	Motomed UE	0.5	10
	Myro	0.3	10
Hand	Reference	1	5
	Gloreha, Yougrabber	0.3	10
Whole body	Reference	1	5
	Nustep	0.3	5
Trunk	Reference	1	5
	Valedo	0.5	10

### Outcomes

For this study, a scoring manual was developed to ensure comparable procedures in each of the participating centers. Characteristics of the included patients (age, sex, BMI, diagnosis, time since stroke, paretic side and cognitive function) were collected at baseline (based on medical records) as recommended by the consensus-based core recommendations from the stroke recovery and rehabilitation roundtable [30]. All co-interventions such as medication or other therapies (e.g., physiotherapy, occupational therapy, psychological counseling, music therapy, water therapy, etc.) during the trial were recorded.

*Feasibility-related outcomes*: The primary goal of this study was to investigate the feasibility of the intensive TAT, which were evaluated by:

- The recruitment process (providing recruitment rate, reasons for exclusion and possibilities to improve this process)
- The feasibility for centers to follow the standardized treatment protocol for the complex highly intensive technology-assisted rehabilitation intervention, and
- Patients’ and therapists’ acceptance of the intervention incorporated, specifically how well the intensive TAT was tolerated.

Intervention-related outcomes were assessed at every training day in terms of devices used and primarily addressed impairment (lower extremity and gait, upper extremity, other) and duration of training. The following outcomes were evaluated:

- Patients’ adherence to the prescribed training was evaluated in terms of scheduled vs. actually performed trainings.
- Subjectively perceived effort of every training was measured with the modified Borg (CR-10 Borg) scale [31].
- The subjectively perceived effectiveness of training series was evaluated with the Patient’s Global Impression of Change Scale (PGICS) [32]. The PGICS consists of two questions, (1) the amount of change on a seven-point scale which reveals the patient’s overall improvement and (2) the degree of change since the beginning of the study on a 11-point scale (0 = much better, 5 = no change and 10 = much worse). A 2-point change on the 11-point scale was considered clinically meaningful [33]. The PGICS was assessed once at the end of the whole intervention.
- Adverse events (AE) were recorded to evaluate safety of the assessments and intervention. An AE was defined as any unintended sign, symptom, or disease in a participant, temporally associated with the intervention. A serious adverse event (SAE) was defined as any event which required a hospital stay and/or resulted in permanent or significant incapacity or disability or an event that was life-threatening. Unrelated AEs (event started in no relationship to intervention) were distinguished from related AEs (event started in relationship to the intervention.

Further outcomes can be categorized into patient-related outcomes and efficiency gains.

For patient-related outcomes an assessment battery was set up, consisting of assessments of all components of the International Classification of Functioning Disability and Health [34], as recommended for robot-assisted exercise trials [30]. Participants were assessed at baseline and at the end of the intervention by self-report or by an assessor according to the type of test.

- Generic functional performance of patients was assessed by the Functional Independence Measure (FIM). The interview-based assessment consists of 18 items, 13 physical domain items and 5 items related to cognition [35]. Based on the level of dependence, each item can be scored from one to seven, with seven indicating complete independence and one indicating complete dependence.
- For assessing upper extremity motor function, the Fugl-Meyer Assessment - Upper Extremity (FMA - UE) published by See et al. [36] was used. The 33-item assessment evaluates the motor function of the arm and the wrist/hand. The FMA-UE has shown good and well-documented psychometric properties, especially validity, and is recommended for assessing motor function in intervention trials [37]. A FMA-UE score below 25 corresponds to severe impairment, indicating that a person is unable to actively move a robot arm without assistance [38].
- Gross manual dexterity was evaluated with the Box&Block Test (BBT) [39]. The test was performed in a sitting position. Patients were instructed to transfer a maximum number of wooden blocks from one compartment to another in 60 s. Both the affected and the unaffected side were assessed and compared with age-related norm scores [40].
- Walking capacity was evaluated with the Walking Index of the Chedoke-McMaster Stroke Assessment (CSMA-WI). The WI is part of the 10-items activity inventory of the CSMA and consists of five items: Walking indoors, walking outdoors (e.g., over rough ground), walking outdoors several blocks, stairs, and the 2-min walk test [41]. All items except for walking distance were rated on a 7-point ordinal scale, where a score of seven indicates complete independence and a score of 1 that full assistance was necessary [42]. Participants’ mobility status was evaluated by using the Functional Ambulation Categories (FAC) assessment developed by Holden et al. (41). Six different categories determine a person’s mobility status and the support needed. A person scoring zero is not able to walk and a person scoring five is fully independent. Usual and maximum gait speed was assessed by the 10- Meter Walking Test (10MWT). Participants had to walk twice the distance of 10 m, first in their usual pace and then in a fast pace (“as fast but as safe as possible”). They were instructed to start two meters in front of the starting line to make sure that gait speed was measured after acceleration.
- Balance abilities were quantified by the performance-based Berg Balance Scale (BBS) [43]. The BBS consists of 14 tasks with a maximum score of 56. A higher scoring indicates better balance. For all tasks, the patient’s best performance was recorded, independently of the hemiparetic leg.
- Participation was assessed using following questionnaires:
  - To assess stroke-specific disabilities and quality of life, the German validated version of the Stroke Impact Scale (SIS, 59 items) was used [44, 45]. The SIS assesses function in eight domains (strength, hand function, (instrumental) ADLs, mobility, memory, communication, emotion, social participation, stroke recovery) and the extent of recovery since stroke onset was quantified on a visual analogue scale [45]. Domain scores were scored on a 5-point Likert scale and domain scores calculated ranging from zero to 100.
  - Health-related quality of life was determined by the five-dimension version of the EQ-5D [46]. The manual published by the EuroQol Group was followed [47]. Patients rated each item on a 5-point level. The results were combined to a health state profile (e.g., 15321) as well as transformed into an index value. For that purpose, the Crosswalk Index Value Calculator was downloaded from the online platform of the EuroQoL Group, and the German value set was used [48]. In addition, the self-related health status was rated on a visual analogue scale (EQ VAS).
- Efficiency gains achieved with the application of technological devices were calculated at the end of the study. Here, trainings with devices were compared to trainings without devices regarding required staff for preparation, training, and wrap-up (Table 2).

Due to the study design, there was no control intervention and therefore no randomization and/or blinding procedures were applied. Centers undertook measures to minimize assessor bias. During final assessment, assessors were kept blind to the baseline values.

### Data analysis

Patient characteristics, training-related data, and feasibility outcomes were analyzed descriptively. Absolute and relative frequency together with parameters of central tendency and distribution were presented.

Due to the small sample size, pre-post analyses of patient-related outcomes were performed by non-parametrical tests. Wilcoxon Signed-rank test was used to examine differences in pretest and posttest scores. Effect size *r* was calculated as *z* statistic divided by square root of the sample size. Additionally, change scores and 95% confidence intervals (CI) were calculated. A 2-sided *p* value of < 0.05 was considered statistically significant. All analyses were performed using the R language for statistical computing (R Foundation for Statistical Computing; Vienne, Austria).

It should be noted that the inferential statistics reported alongside the estimation of intervention effects should be considered exploratory. The findings are not meant to represent the results of a definite trial nor will they serve as a basis for sample size calculations for a main trial [49].

For the efficiency gain analysis, staff time was presented relative to regular therapy without the respective device.

## Results

### Enrolment and patient characteristics

**Fig. 1:**
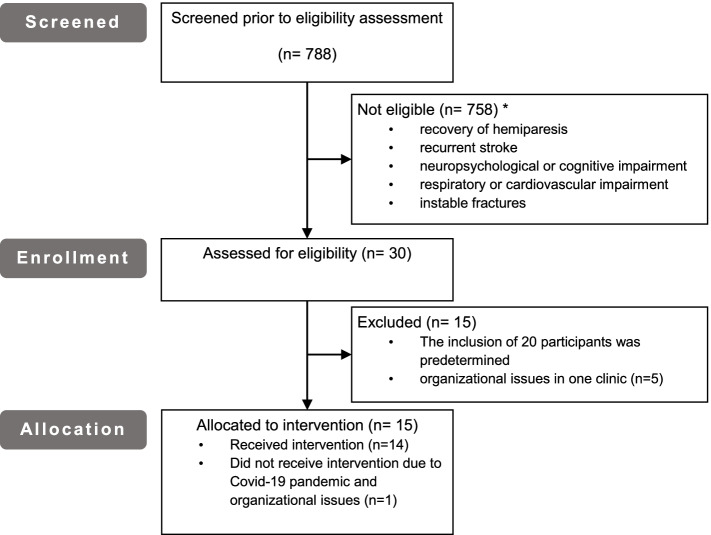
Screening and enrolment of study participants

Patients were screened and recruited between November 2018 and November 2019 at the four participating centers (Fig. 1 screening and enrolment). Participating patients were diagnosed with stroke between 2 months to 9 years prior to recruitment for the study. Due to a reorganization, one clinic was no longer able to participate.

Fourteen patients were included in the study and received the intervention at the end of their first inpatient rehabilitation period or in an outpatient setting. Due to the COVID-19 pandemic, one patient in one clinic could not complete all training sessions. Patients’ characteristics are provided in Table 3.

**Table 3:** Patients’ characteristics

Characteristic	*n* = 14
**Age,** years, mean ± SD	60.8 ± 12.5
**Sex, ** *n* (%)	
Female	4 (28.6%)
Male	10 (71.4%)
**Body mass index (BMI)**, mean ± SD	26.2 ± 4.1
**Diagnosis, ** *n* (%)	
Ischemic stroke	10 (71.4%)
Intracranial hemorrhage	4 (28.6%)
**Paretic side, ** *n* (%)	
Left	9 (64.3%)
Right	5 (35.7%)
**Montreal Cognitive Assessment** (MoCA), 0-30, mean ± SD	24.9 ± 2.8

### Intervention-related data

#### Devices used and duration of training

All 14 patients exercised between twelve and 21 days with technological devices. They received between 28 and 82 (mean 46 ± 15) TATs during the study period, which corresponded to two to seven daily interventions. In total, 652 interventions with 20 different devices were undertaken (Table 4). Most trainings were scheduled to either up to 30 min (*n* = 382) or 31–45 min (*n* = 210, Table 4).

**Table 4:** Number of devices with respective number of interventions

Impairments addressed	Number of devices used	Number of interventions (total = 652)
Lower extremity and gait	10	299
Upper extremity	9	201
Other	1	152

#### Patients’ adherence

**Fig. 2:**
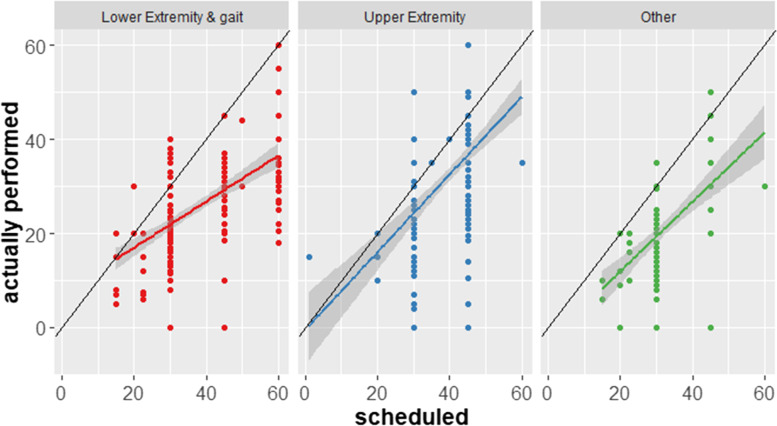
Trainings with technological devices (*n* = 646) in minutes scheduled vs. actually performed

In general, patients showed a high adherence to their scheduled TAT (Table 5). The number of scheduled and performed trainings correlated with *r* = 0.56, 95% CI: 0.50–0.61. Adherence rates for different impairments are shown in Fig. 2.

**Table 5:** Comparison between scheduled and performed trainings

	Scheduled				
Performed *n* (% of scheduled)	≤ 15 min	16–30 min	31–45 min	> 45 min	Sum
**≤ 15 min**	**7 (87.5)**	87 (22.8)	5 (2.4)	0	98
**16–30 min**	1 (12.5)	**241 (63.1)**	58 (27.6)	22 (47.8)	322
**31–45 min**	0	31 (8.1)	**132 (62.9)**	19 (41.3)	182
**> 45 min**	0	1 (0.3)	11 (5.2)	**5 (10.9)**	17
**Did not take place**	0	22 (5.8)	4 (1.9)	0	26
**Sum (%)**	8 (100)	382 (100)	210 (100)	46 (100)	646

In bold: trainings performed as scheduled, missing *n* = 6

#### Perceived exertion (modified Borg scale)

**Fig. 3:**
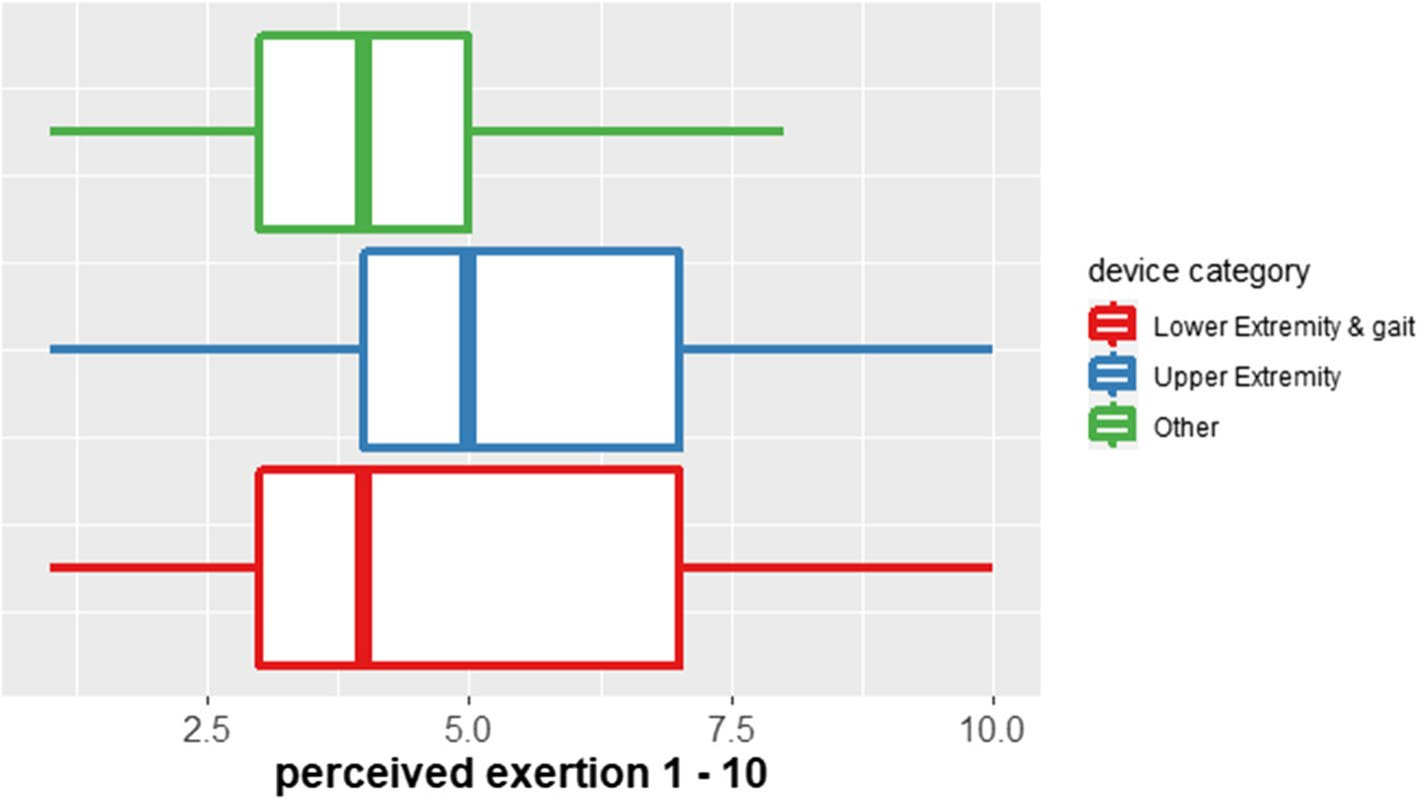
Perceived exertion during trainings

The median perceived exertion on the CR-10 Borg scale during all trainings amounted to 4 (IQR: 3–6). Patients with different impairments reported only small differences of their perceived exertion (Fig. 3).

#### Adverse events

No serious adverse event was observed. However, 33 adverse events (AE) were reported during or after 652 trainings (5.1%). The AEs were equally distributed over device categories or impairments with 14 occurring during lower extremity and gait trainings (in 4.7% of the trainings), 11 during upper extremity trainings (in 5.5% of the trainings), and 8 during other trainings (in 5.3% of trainings) according to the number of trainings (Table 4).

The most frequent symptoms were tiredness and exertion (*n* = 17) or pain (*n* = 14). Two AEs were unrelated to the intervention but were allocated by therapists to lower extremity and gait trainings.

#### Subjectively perceived effectiveness of training

Meaningful improvement («much improved» or «very much improved») regarding the perceived effectiveness of training series, measured by the PGICS, was reported by 50% of the PaS. A clinically meaningful change was indicated by 35.7% of the PaS.

### Patient-related outcomes

Table 6 presents the data on patient-related outcomes for pre- and post-intervention assessment values.

**Table 6:** Patient-related outcomes pre- and post-intervention

	Pre-intervention	Post-intervention	Median change [95% CI]	***p*** (α = 0.05)	***r*** [95% CI]
**Lower extremity, gait, and balance**					
10MWT (in seconds) comfortable (*N* = 12)	28.6 [22.9]	19.0 [19.6]	− 1.75 [− 8.4 to − 0.6]	0.014	0.738 [0.38–0.89]
10MWT (in seconds) maximum (*N* = 12)	21.6 [23.4]	15.1 [19.3]	− 1.25 [− 9.5 to − 0.9]	0.016	0.679 [0.29–0.89]
CSMA-WI, walking capacity	16 [8.3]	19 (7.8)	2 [0.0–3.5]	NS	–
BBS, balance abilities	32 [24]	39 [18.8]	5.5 [2.0–9.0]	0.001	0.88 [0.88–0.89]
FAC, mobility status	2 [2]	3.5 (3)	0.5 [0.0–1.0]	0.015	0.703 [0.46–0.86]
**Upper extremity**					
FMA-UE, upper extremity motor function	16 [12.5]	27.5 [23.8]	8 [3.0–12.0]	0.002	0.87 [0.84–0.91]
BBT, gross manual dexterity (affected side)	0 [19.8]	1 [30.8]	1 [0.0–8.0]	0.014	0.74 [0.53–0.86]
**Generic outcome measures**					
SIS, stroke impact (64–320)	219 [39.8]	238 [38]	13 [0.0–39.0]	0.003	0.78 [0.54–0.88]
SIS, stroke recovery (0–100%)	50 [35]	65.5 [17.5]	7.5 [− 5.0 to 30.0]	NS	–
FIM, generic functional performance	102 [47.5]	104.5 [32.5]	5.0 [1.0–13.0]	0.002	0.86 [0.78–0.89]
EQ-5D Index	0.7 [0.16]	0.8 [0.14]	0.09 [0.0–0.1]	NS	–

Sampling units: *N* = 14; *CI* confidence interval, *P p*-value provided by Wilcoxon signed-rank test, *NS* non-significant, *r* effect size *r*, calculated as *z* statistic extracted from a paired-sample Wilcoxon signed-rank test, divided by square root of the sample size, *10MWT* 10 Meter Walking Test, *CSMA-WI* Chedoke-McMaster Stroke Assessment – Walking Index, *BBS* Berg Balance Scale, *FAC* Functional Ambulation Category, *FMA-UE* Fugl-Meyer Assessment -Upper Extremity, *BBT* Box&Block Test, *SIS* Stroke Impact Scale, *FIM* Functional Independence Measure

#### Effect of technology-assisted training on lower extremity and gait

After receiving TAT, participants improved their mean time for the 10MWT from 25.2 s (SD 23.8) during pre-assessment to 21.2 s (SD 15.7) during post-assessment at maximum speed (*p* = 0.016, *r* = 0.68). Although statistical analysis was based on the variability of ranks, interpretation of mobility status measured by the FAC should be based on the raw scores. Pre- and post-training median FAC scores improved from 2 to 3.5. A FAC score of two means that participants required assistance from another person in the form of continuous or intermittent manual contact for walking, a FAC score of 3.5 indicates unrestricted walking with only verbal supervision. Walking capacity, monitored by the CSMA-WI, did not show statistically significant improvements.

#### Effect of technology-assisted training on upper extremity

The participants’ median scores of FMA-UE increased from 16 (IQR 12.5) at baseline to 27.5 (IQR 23.8) after TAT. Statistical analyses indicate significant improvements of both measurements for upper extremity performance (FMA-UE *p* = 0.002, *r* = 0.87; BBT *p* = 0.014, *r* = 0.74).

#### Effect of technology-assisted training on generic functional performance and quality of life

Regarding generic functional performance, participants achieved a median 5-point change on the FIM. Improvements relating to QoL were visible in the SIS (*p* = 0.003, *r* = 0.78) but not in the EQ-5D Index and the corresponding SIS stroke recovery item.

### Cost-specific outcomes

**Fig. 4:**
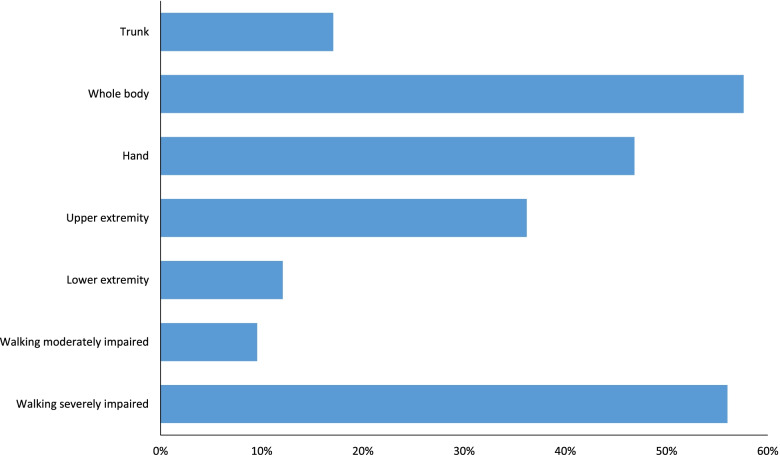
Efficiency gain in % staff time. The reference relates to the same trainings without devices

Pertaining to staff-time, TATs were more efficient. Depending on the training goal, the efficiency gains amounted between 10% and 58% (Fig. 4).

## Discussion

In this study, the feasibility of a highly intensive TAT was evaluated in a longitudinal single-group design to evaluate the safety and acceptance of the intervention and to establish its feasibility for a subsequent RCT. Fourteen patients in the subacute and chronic phase after stroke received four weeks of TAT with three to five training days per week in addition to conventional treatment in four neurological rehabilitation centers.

Technology-assisted motor training was implemented in neurological rehabilitation based on theoretical rationale and fundamental research [50]. An important element of the technological assistance is the possibility to train at high intensities, i.e., number of repetitions. Evidence about the effectiveness of TAT for motor rehabilitation becomes established only gradually in an ongoing process. The present study presents a first attempt to address the feasibility of TAT in PaS, who had completed their primary rehabilitation using extraordinarily high intensities as defined by three to five weekly training days each comprising at least five trainings with a duration of 45 min. The duration of this TAT was set at 4 weeks. This duration was considered by consensus to be adequate to evaluate the feasibility of the program. In addition, it was a reasonable time for participating centers to provide equipment and staff. According to the literature, a training duration of at least 2 weeks might be adequate to elicit functional responses. The duration of most repetitive task trainings (84%) included in the Cochrane Review by French et al. [51] was up to 12 weeks. However, 19 of 33 studies (58%) reported a training duration equal to our study, i.e., 4 weeks. The study revealed that only a small fraction of screened patients met the inclusion criteria and consented for participation. The results of the PaS, who participated, suggest that highly intensive TAT is feasible in an inpatient as well as an outpatient setting. Participants attended most scheduled trainings and reported only a low number of undesired events. Besides, the study results indicate improvements in multiple measures of functional performance after highly intensive TAT.

### Technology-assisted training

There is a substantial amount of literature concerning the efficacy of TAT and the importance of the therapy dosage in the rehabilitation of stroke patients [23, 52, 53]. Our data confirm these previous results. High-intensity TAT led to clinically meaningful improvements in functional scores as well as in the self-perceived effectiveness, captured by the PGICS. Due to the uncontrolled design of this feasibility study, only an intra-group comparison before and after the 4 weeks of highly intensive TAT was possible. Therefore, we cannot exclude that patients would have equally improved if they had received the same dosage of conventional therapies. Nonetheless, the results are encouraging and should be studied in an adequately powered RCT using rater-blinded assessments.

### Feasibility

In the present study, less than 4% of the screened patients finally participated. This is at least partially due to the rigorous inclusion and exclusion criteria. Many patients were excluded because of recurrent stroke, but also because of the absence of residual hemiparesis. A relatively high proportion of patients could not participate due to cardiovascular, respiratory, or orthopedic disease or cognitive impairment. In addition, specifically for outpatients, logistical challenges represented a barrier. The slow recruitment rate persisted even after adapting the inclusion criteria. Given the selected sample, we cannot exclude that highly intensive TAT may be applicable only in a subset of patients without clinically relevant comorbidities or cognitive dysfunction. It appears likely that highly intensive TAT is rather feasible in PaS with an otherwise good general health and preserved cognitive function. Additional studies should investigate to what degree high-intensity TAT is feasible in medically more compromised patients. It can also be hypothesized that highly intensive TAT may be useful in patients with recurrent stroke. Low drop-out rates during the intervention phase however support the assumption that high-intensity TAT is acceptable to PaS and in about the range of comparable studies [54].

### Recommendations for further studies

Highly intensive TAT in the present study included a variety of different devices in four different clinics suggesting that this therapy approach could be feasible for a wide range of health care providers. However, the specialized equipment also requires adequately trained personnel in order to set up therapy plans fulfilling the criteria for highly intensive TAT as used in this trial. Current rehabilitation devices unfold their therapeutic potential only if they are used in conjunction with skilled therapeutic staff [50, 55]. Interestingly, the study could be successfully performed under in- and outpatient conditions. This might be attributable to the relatively good health condition of the sample, which was also considered essential for other outpatient rehabilitation programs [56]. The experimental training of this study involved several hours of practicing with the help of technological devices. To keep up motivation has been considered challenging [57]. Participants of the present study attended between 5 and 87.5% of the planned sessions. Lower adherence rates were observed with trainings with a longer duration than 45 min. It is therefore recommended for future trials involving high-intensity TAT to consider the length of training sessions.

The core of this study was intensive motor training in order to maximize functional improvements. The optimal intensity however depends on the actual condition of the participants and needs to be adapted on a regular basis [58]. Here, the degree of exertion was measured by the modified Borg scale, which was acceptable to participants. However, the results revealed a broad ranging from 1 to 10, indicating that not all trainings have been performed at a challenging intensity. Possible explanations may be that participants did not train at their limit during the first sessions, when they still familiarized with the devices, or that they rated later training sessions less strenuous once used to the devices and training. The safety profile with a relatively low proportion of adverse events in relation to the total number of sessions was to be expected and is in line with other reports [59, 60]. No serious adverse events did occur. Adverse events mostly referred to exertion and tiredness or pain and were always transitory. The relative high proportion of tiredness/exertion among the adverse events raises the question whether the modified Borg scale alone can adequately capture cognitive fatigue besides physical exertion. The use of an additional instrument may be warranted. Overall, the study provides evidence that highly intensive TAT is safe in patients in the subacute or chronic phase after a stroke.

### Study strengths and limitations

The present study opens new avenues for a more effective rehabilitation of PaS even in outpatient settings. Considering the importance of highly repetitive and intensive therapy programs for the improvement of sensorimotor impairment after stroke, the employment of TAT may facilitate the access of PaS to individualized rehabilitation at adequate intensities.

TAT is associated with a slightly increased preparation time for each session as compared with conventional therapies. However, overall TAT was associated with significant efficiency gains, which resulted in the benefit that higher training intensities could be provided with about the same number of personnel. This means that TAT can be performed with a lower therapist-to-patient ratio than conventional therapies. This preliminary result needs to be confirmed with a sound cost-effectiveness study, since TAT is usually not adequately reimbursed by health insurances and other cost bearers.

There are limitations to this study. Apart from the single-group design and the low ratio of recruited-to-screened patients, one main limitation of the present study was the small sample size. Unfortunately, the envisaged number of 20 patients could not be reached because of the aforementioned recruitment issues, organizational restructuring in one clinic, and the beginning of the COVID-19 pandemic towards the end of the trial. We can therefore not exclude that the low number of participants may have biased the results of the present study.

The calculation of efficiency gains was based on reference values estimated by expert clinicians rather than empirical data. Therefore, efficiency gains observed in this study might be slightly be under- or overestimated. Additionally, not at all study centers assessors were blinded pertaining to the aims of the study aims, which may have led to biased estimates of patient-related outcomes.

## Conclusions

This study proposes that highly intensive TAT is feasible for in- and outpatients with subacute or chronic stroke. Highly intensive TAT seems to be effective but may be applicable only in a subset of PaS and presenting with only minor cognitive impairment and in a condition of good general health. Further clinical trials are warranted in order to define the most comprehensive approach to highly intensive TAT and to investigate its efficacy in patients with chronic neurological disorders.

## Data Availability

The datasets used/analyzed during the current study are available from the corresponding author on reasonable request.

## Abbreviations

10mWT: 10-meter walk test
BBS: Berg Balance Scale
BBT: Box and Block Test
CMSA: Chedoke-McMaster Stroke Assessment Measure
EQ-5D: EuroQol-5D
FAC: Functional Ambulation Categories
FIM: Functional Independence Measurement
SIS: Stroke Impact Scale
TAT: Technology-assisted training
PGIC: Patient Global Impression of Change
